# A computed tomography scoring system to assess pulmonary disease among premature infants

**DOI:** 10.1590/S1516-31802010000600004

**Published:** 2010-12-02

**Authors:** Márcia Cristina Bastos Boechat, Rosane Reis de Mello, Kátia Silveira da Silva, Pedro Daltro, Edson Marchiori, Eloane Guimarães Ramos, Maria Virgínia Peixoto Dutra

**Affiliations:** I MD. Pediatric radiologist, Instituto Fernandes Figueira (IFF), Fundação Oswaldo Cruz (Fiocruz), Flamengo, Rio de Janeiro, Brazil.; II MD. Neonatologist, Instituto Fernandes Figueira (IFF), Fundação Oswaldo Cruz (Fiocruz), Flamengo, Rio de Janeiro, Brazil.; III MD. Epidemiologist, Instituto Fernandes Figueira (IFF), Fundação Oswaldo Cruz (Fiocruz), Flamengo, Rio de Janeiro, Brazil.; IV MD. Radiologist, Universidade Federal Fluminense (UFF), Niterói, Rio de Janeiro, Brazil.; V BSc. Engineer and statistician, Instituto Fernandes Figueira (IFF), Fundação Oswaldo Cruz (Fiocruz), Rio de Janeiro, Brazil.

**Keywords:** Tomography, Tomography, X-ray computed, Infant, premature, Lung, Bronchopulmonary dysplasia, Tomografia, Tomografia computadorizada por raios X, Prematuro, Pulmão, Displasia broncopulmonar

## Abstract

**CONTEXT AND Objective:**

High-resolution computed tomography (HRCT) is considered to be the best method for detailed pulmonary evaluation. The aim here was to describe a scoring system based on abnormalities identified on HRCT among premature infants, and measure the predictive validity of the score in relation to respiratory morbidity during the first year of life.

**DESIGN AND SETTING:**

Prospective cohort study in Instituto Fernandes Figueira, Fundação Oswaldo Cruz.

**Methods:**

Scoring system based on HRCT abnormalities among premature newborns. The affected lung area was quantified according to the number of compromised lobes, in addition to bilateral pulmonary involvement. Two radiologists applied the score to 86 HRCT scans. Intraobserver and interobserver agreement were analyzed. The score properties were calculated in relation to predictions of respiratory morbidity during the first year of life.

**Results:**

Most of the patients (85%) presented abnormalities on HRCT, and among these, 56.2% presented respiratory morbidity during the first year of life. Scores ranged from zero to 12. There was good agreement between observers (intraclass correlation coefficient, ICC = 0.86, confidence interval, CI: 0.64-0.83). The predictive scores were as follows: positive predictive value 81.8%, negative predictive value 56.3%, sensitivity 39.1%, and specificity 90.0%.

**Conclusion:**

The scoring system is reproducible, easy to apply and allows HRCT comparisons among premature infants, by identifying patients with greater likelihood of respiratory morbidity during the first year of life. Its use will enable HRCT comparisons among premature infants with different risk factors for respiratory morbidity.

## RESUMO

**CONTEXTO E Objetivo:**

Tomografia computadorizada de alta resolução (TCAR) é considerada o melhor método para avaliação pulmonar detalhada. O objetivo foi descrever um sistema de escore baseado em alterações identificadas nas TCAR de lactentes prematuros e medir a validade preditiva do escore em relação à morbidade respiratória no primeiro ano de vida.

**TIPO DE ESTUDO E LOCAL:**

Estudo de coorte prospectiva no Instituto Fernandes Figueira, Fundação Oswaldo Cruz.

**Métodos:**

Sistema de escore baseado em alterações nas TCAR de lactentes prematuros. A área pulmonar alterada foi quantificada conforme o número de lobos alterados, acrescido do comprometimento pulmonar bilateral. Dois radiologistas aplicaram o escore em 86 TCAR. Foram analisadas as confiabilidades intraobservador e interobservador e calculadas as propriedades do escore em relação à predição da morbidade respiratória no primeiro ano de vida.

**Resultados:**

A maioria (85%) dos pacientes apresentou TCAR anormal, e dentre estes, 56,2% apresentaram morbidade respiratória no primeiro ano de vida. Valores do escore variaram de zero a 12. Houve boa concordância entre os observadores (coeficiente de correlação intraclasse, CCI = 0,86, intervalo de confiança, IC: 0,64-0,83). Os valores preditivos do escore foram: valor preditivo positivo 81,8%, valor preditivo negativo 56,3%, sensibilidade 39,1% e especificidade 90,0%.

**Conclusão:**

O sistema de escore é reprodutível, de fácil aplicação e permite a comparação de TCAR de pacientes prematuros, identificando pacientes com maior probabilidade de morbidade respiratória no primeiro de vida. Seu uso permitirá a comparação de TC de lactentes prematuros com diferentes fatores de risco para morbidade respiratória.

## INTRODUCTION

Over the last decade, evolution in neonatal intensive care and the use of prenatal steroid therapy and surfactant treatment have contributed greatly towards extending the survival of extremely premature and/or very low birth weight newborns. New methods of mechanical ventilation and the entire therapeutic armamentarium used among these infants have helped decrease the severity of neonatal respiratory distress syndrome, but they have not prevented the development of pulmonary disease.^1^

Some 30% of infants with birth weight less than 1200 g who receive oxygen therapy and mechanical ventilation for extended periods develop bronchopulmonary dysplasia (BPD), which is considered to be the principal cause of chronic lung disease in childhood.^1^ BPD is associated with high morbidity and mortality rates,^2^ especially during the first two years of life, as demonstrated by the persistence of respiratory symptoms and the higher number of hospitalizations among these patients, compared with premature infants without BPD.^3^ Over the course of childhood, there is a trend towards improvement in both lung function and respiratory symptoms, although residual abnormalities can persist into adolescence and young adulthood,^3-5^ along with residual pulmonary anomalies on chest radiography and computed tomography (CT).^2,6,7^

Chest radiographic abnormalities among premature newborns receiving mechanical ventilation and oxygen therapy who develop lung disease do not reflect the degree of pulmonary involvement. On the other hand, high-resolution computed tomography (HRCT) is considered to be the best imaging method for detailed evaluation of the pulmonary parenchyma.^8^ Chest CT is frequently ordered for premature infants with respiratory symptoms. However, there are few specific studies on the role of this test among premature infants, especially during the neonatal period and the first year of life. Most of the existing studies did not standardize the interpretation of CT findings among cases of neonatal pulmonary disease or did not evaluate the predictive value of these abnormalities in relation to the morbidity that these patients may present during childhood, especially during the first year of life.^2,5,8-10^ CT scores for evaluating pulmonary involvement, specifically among premature newborns with BPD, were recently developed.^7,11^

## OBJECTIVE

The objectives of this study were to describe a new scoring system based on the pulmonary morphological abnormalities identified on HRCT among premature infants during the neonatal period and to measure its predictive validity in relation to respiratory morbidity during the first year of life.

## MATERIAL AND METHODS

The scoring system developed in this study was applied to HRCT scans on 86 premature infants born between January 1, 1998, and August 31, 2000, who were admitted to a neonatal intensive care unit (NICU) in a maternity and children's hospital in the city of Rio de Janeiro, Brazil. The study was approved by the Research Ethics Committee of Instituto Fernandes Figueira.

All the infants were part of a prospective cohort study that evaluated respiratory morbidity during the first year of life among very low birth weight premature newborns. Premature infants with gestational age less than 34 weeks whose birth weight was either less than 1500 g or appropriate for gestational age, and who underwent HRCT scans, were included. The exclusion criteria were congenital malformations, congenital infections and genetic syndromes. The population in this study represented a convenience sample.

HRCT was performed shortly before hospital discharge on clinically stable infants breathing room air. All the scans were performed using ProSpeed-S™ (General Electric, Milwaukee, United States), with slices of 1 mm in thickness at intervals of 10 mm to 15 mm (six to nine slices per test), with settings of 90 mAs and 120 kV, without sedation, and with the patient preferably sleeping spontaneously after feeding.

This scoring system was developed on the basis of the radiographic scoring system^12-16^ and CT scoring system^7,17^ for BPD and on the radiographic scoring system^18-21^ and CT scoring system^22-26^ for cystic fibrosis, which is the most widely studied chronic lung disease in childhood.

**Figure 1.**
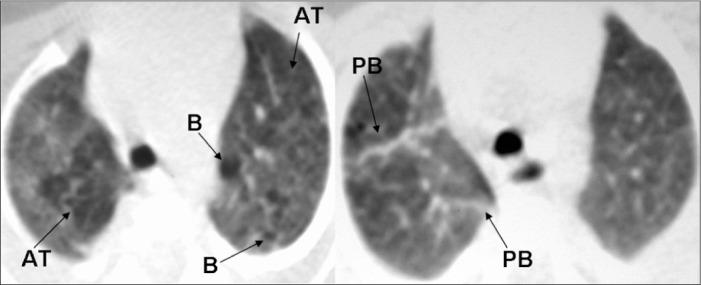
High-resolution computed tomography (HRCT) scan showing air trapping (AT), air bubbles (B) and parenchymal band (PB).

**Figure 2.**
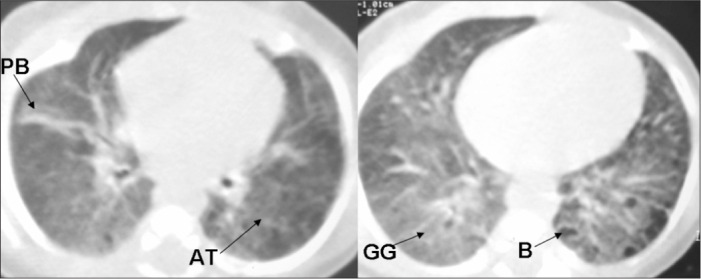
High-resolution computed tomography (HRCT) scan showing air trapping (AT), air bubbles (B), ground-glass opacity (GG) and parenchymal band (PB).

**Figure 3.**
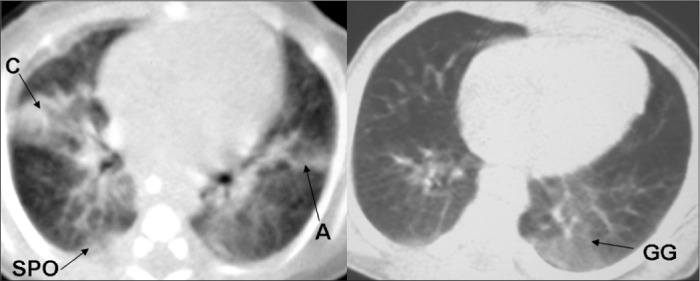
High-resolution computed tomography (HRCT) scan showing atelectasis (A), consolidation (C), subpleural opacity (SPO) and ground-glass opacity (GG).

The accumulated experience of evaluating HRCT scans on children with pulmonary disease in a public maternity and children's hospital, based on lesions described in the specific literature^2,5-8,10,11,17^ made it possible to identify the principal abnormalities found using chest CT among premature infants. Webb et al.,^27^ Lucaya and Le Pointe^28^ and Hansell et al.^29^ characterized the following CT abnormalities among premature newborns: atelectasis (opacity with reduced lung volume, secondary to alveolar collapse); consolidation (opacity, expressed as increased density of the pulmonary parenchyma, usually homogeneous, accompanied by obscuration of the underlying blood vessels); ground-glass opacity (increased density of the pulmonary parenchyma without obscuration of the vessels); air trapping (areas with decreased attenuation interspersed with areas of normal attenuation); and air bubbles (lesion containing air, with thin, well-defined walls, not always possible to distinguish from pulmonary cyst). Figures 1, 2, and 3 illustrate the abnormalities comprising the scoring system.

In addition to identifying the abovementioned lung abnormalities, this scoring system evaluated the topographic site (pulmonary lobes) and quantified the affected lung area by means of a score attributed to the lobes affected by the abnormalities, with the addition of two more points in the case of bilateral lung involvement. In this study, the lingula was defined as a separate lobe and not as part of the left upper lobe.^11^ Each of the five abnormalities (atelectasis, consolidation, ground-glass opacity, air trapping and air bubbles) received a score from zero to six. As already stated, two more points were added in the case of bilateral lung involvement. Score zero was assigned to cases of absence of abnormalities, and the other values were assigned according to the number of lobes affected by abnormalities (one point per lobe). In the case of unilateral involvement, scores from one to three were assigned. For bilateral involvement, these scores ranged from four to six, depending on the number of abnormal lobes. Thus, for each of the abnormalities, a score could be assigned from zero (absent) to eight, corresponding to values from one to three for unilateral involvement and four to eight for bilateral involvement. The maximum total score in the proposed scoring system was 40 points, i.e. eight points for each of the five CT abnormalities, such that the highest values represented the most heavily altered scans and the lowest values represented the least altered scans.

Two pediatric radiologists (BM, DP) with more than 10 years’ experience had evaluated the CT scans previously, when there was still no scoring system, and they now conducted the intra and interobserver reliability study for each lesion identified.^2^ For the purposes of the current study, these same radiologists jointly identified the target abnormalities and underwent training to apply the scoring system. Subsequently, the two observers independently applied the scoring system to the 86 HRCT scans, blinded to the clinical data and with no access to their own and each other's previous readings.

The principal observer (BM) applied the scoring system to all 86 HRCT scans on two different occasions, with a three-month interval between the two. The intra and interobserver reliability of the scores was evaluated by means of the intraclass correlation coefficient (ICC)^30^ and the Bland-Altman method.^31^

All the infants were followed up at the high-risk neonatal outpatient clinic by a pediatrician who was unaware of the HRCT results. Respiratory evolution during the first year of life was evaluated by means of physical examination, and the presence of respiratory complications (persistent wheezing, and/or hospitalization, and/or pneumonia) during the intervals between consultations was recorded. The consultations were held monthly or according to clinical need.^2^ Data were collected at each consultation, and upon completing 12 months of corrected age, it was determined whether the child had presented respiratory morbidity, defined as the presence of one or more of the following: persistent wheezing (presence of two or more episodes of wheezing causing respiratory difficulty, observed by the pediatrician by means of pulmonary auscultation, and which required bronchodilatory medication); hospitalization due to respiratory problems (hospitalization of the infant for more than 24 hours); or pneumonia (presence of tachypnea, intercostal and subcostal retraction, crackles and proven radiological abnormalities; chest X-rays were interpreted by a pediatric radiologist).^2^

The scores were correlated with respiratory morbidity variables. To assess the score characteristics (sensitivity and specificity) and predictive validity (positive predictive value [PPV] and negative predictive value [NPV]) in relation to respiratory morbidity, a receiver operating characteristic (ROC) curve was constructed that indicated the best cutoff point according to the greatest area under the curve. A statistical test was also performed (α < 0.05) to find out whether the area under the curve was significantly greater than 50%. The likelihood ratio was also calculated for the positive tests (score above the cutoff) and negative tests (score below the cutoff).

Epi Info™ version 3.32 was used to construct the database. The statistical analyses used the softwares Epi Info 3.32, Statistical Package for Social Sciences (SPSS) version 12.0 and MedCalc version 9.3.8.0.

## RESULTS

During the abovementioned period, 179 premature newborns were admitted to the NICU with gestational age at birth ranging from 23 to 33 weeks (mean of 28 weeks, standard deviation, SD: 2.3 weeks) and birth weight ranging from 610 to 1480 g (mean of 1101 g, SD: 235 g). Among these premature infants, 20 (11.17%) evolved to death and 58 (32.4%) were excluded (41 small for gestational age, seven with congenital malformations, seven with genetic syndrome and three with congenital infections). The parents of four infants (2.23%) refused to allow their participation in the study, and 11 patients (6.14%) failed to undergo HRCT scans due to technical problems in the CT equipment. This left 86 patients who underwent HRCT scans, with gestational ages corrected for prematurity ranging from 30 to 40 weeks (mean of 36 weeks, SD: 2 weeks) and chronological mean age of 59 days (SD: 26 days) at the time of the scan. Among these, 24 (27.9%) met the clinical diagnostic criteria for BPD (defined as the need for supplemental oxygen for 28 days or more).^32^ All the premature infants who underwent HRCT scans were followed up throughout their first year of life. There was no attrition rate regarding the outcome of respiratory morbidity. Out of the 86 patients, 73 (85%) showed abnormalities on HRCT scans. Among the latter, 56.2% presented respiratory morbidity during the first year of life.

A mean score was assigned to each CT by each radiologist. Table 1 shows the mean scores for the single pulmonary abnormalities, and the final score assigned by the observers can be seen in the last column. According to the criteria, 15% of the patients had a normal CT (mean score = zero). The mean score of the CT tests ranged from 0.5 to 12 (mean 4.2; median 3.5). The most frequent CT abnormalities were atelectasis (80.2%) and ground-glass opacity (50%).

**Table 1.** Mean scores for separate computed tomography (CT) findings and total score, as evaluated by the two observers from high-resolution computed tomography (HRCT) scans on 86 premature newborns, and the mean score from the evaluations

CT findings (possible range of scores)	A1 Mean value (minimum-maximum)	A2 Mean value (minimum-maximum)	B Mean value (minimum-maximum)	Mean score Mean value(minimum-maximum)
Atelectasis (0-8)	2.73 (0-8)	2.42 (0-8)	3.08 (0-8)	2.75 (0-8)
Ground-glass opacity (0-8)	0.67 (0-6)	0.51 (0-6)	0.89 (0-7)	0.70 (0-6.5)
Air trapping (0-8)	0.14 (0-4)	0.16 (0-4)	0.22 (0-4)	0.19 (0-2.5)
Air bubbles (0-8)	0.36 (0-6)	0.40 (0-5)	0.40 (0-4)	0.40 (0-4.5)
Consolidation (0-8)	0.07 (0-4)	0.07 (0-4)	0.25 (0-5)	0.16 (0-4.5)
Total score (0-40)	3.98 (0-15)	3.56 (0-12)	4.81 (0-14)	4.18 (0-12)

A1 = Principal observer's first evaluation; A2 = Principal observer's second evaluation; B = Second observer's evaluation.

Assessment of intraobserver reliability was based on the two evaluations by the principal observer, while interobserver reliability was based on the second evaluation by the principal observer and the evaluation by the second observer. Table 2 shows the results from the intraobserver and interobserver reliability evaluations for the total score and single CT abnormalities. The ICC for intraobserver reliability was very low for air trapping (ICC 0.26). For the other CT findings, the ICC ranged from 0.73 to 0.97, showing very good agreement. For interobserver reliability, the ICC ranged from 0.45 to 0.86.

**Table 2.** Intraclass correlation coefficient (ICC) for intraobserver and interobserver reliability in relation to total score and separate computed tomography (CT) findings

CT findings	Intraobserver reliability	Interobserver reliability
Atelectasis	0.87 (CI 0.81-0.91)	0.84 (CI 0.76-0.90)
Ground-glass opacity	0.73	0.73
	(CI 0.61-0.81)	(CI 0.59-0.83)
Air trapping	0.26	0.45
	(CI 0.06-0.45)	(CI 0.16-0.64)
Air bubbles	0.95	0.60
	(CI 0.91-0.96)	(CI 0.38-0.74)
Consolidation	0.97	0.68
	(CI 0.95-0.98)	(CI 0.50-0.79)
Total score	0.89	0.86
	(CI 0.84-0.93)	(CI 0.78-0.90)

CI = 95% confidence interval.

**Figure 4.**
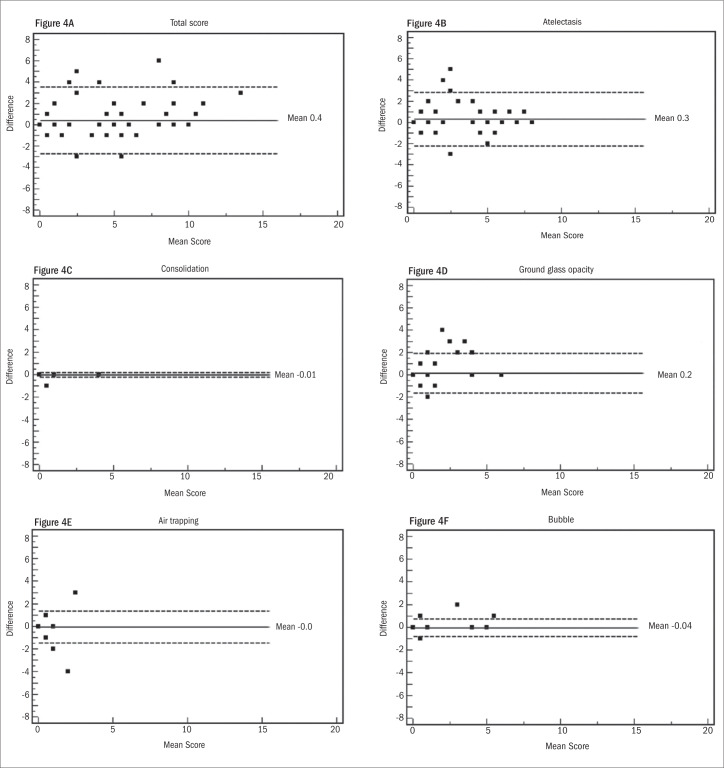
Bland-Altman plots showing variance in intraobserver reproducibility in relation to total computed tomography (CT) score and CT findings. Horizontal lines show the mean and ± 2 SD (standard deviation). The Y-axis shows the difference in mean score between first and second observations.

**Figure 5.**
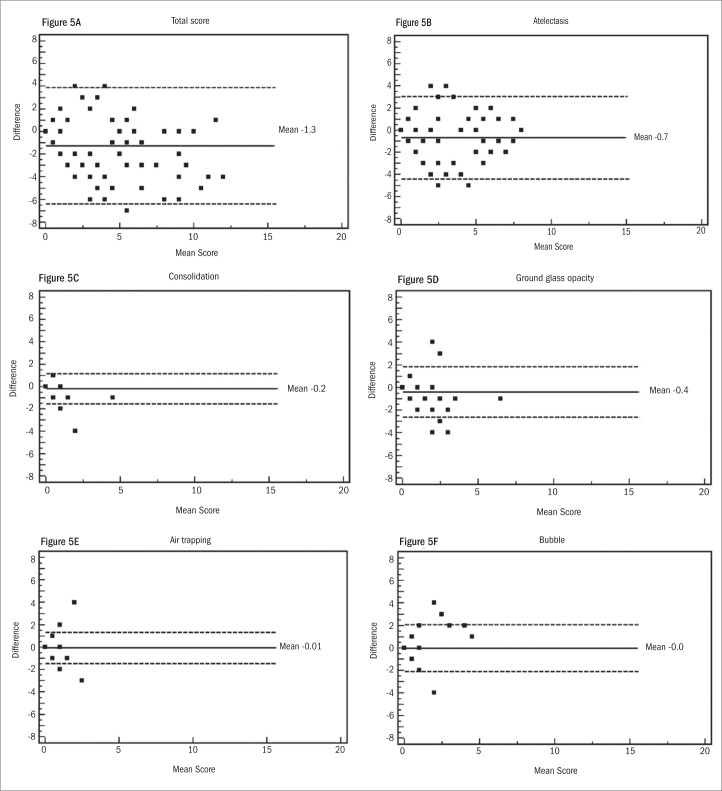
Bland-Altman plots show variance in interobserver agreement in relation to total computed tomography (CT) score and CT findings. Horizontal lines show the mean and ± 2 SD (standard deviation). The Y-axis shows the difference in mean score between two observers.

Intra and interobserver reliability values for the total score and single CT abnormalities were also evaluated in accordance with the Bland-Altman method, as shown in Figures 4 and 5. For intraobserver reliability, the total score showed a trend towards lower agreement at the higher values (Figure 4A), and no significant difference was observed between the results from the two evaluations in relation to consolidation (Figure 4C), ground-glass opacity (Figure 4D) or air bubbles (Figure 4F). The graphical method showed a larger difference between the first and second evaluations for atelectasis (Figure 4B) and showed no difference for air trapping (Figure 4E).

The interobserver evaluation using the Bland-Altman method showed a mean difference of −1.3 in the total score. The differences ranged from −6 to 4 and did not show any trend (Figure 5A). For consolidation, air trapping and air bubbles, the differences between the evaluations by the two radiologists were minimal (Figures 5C, 5E and 5F, respectively). The differences were 0.7 for atelectasis (Figure 5B) and 0.4 for ground-glass opacity (Figure 5D).

To measure the predictive validity of the scoring system in relation to respiratory morbidity over the first year of life, a receiver operating characteristic (ROC) curve was constructed, based on the scores and the presence of respiratory morbidity. The criterion chosen for the best cutoff point was a score greater than six, with an area under the curve of 0.65% (95% confidence interval, CI 0.54-0.75). This cutoff (score > 6) showed the best relationship between sensitivity (39.1%; 95% CI 25.1-54.6), specificity (90.0; 95% CI 76.3-97.1), positive likelihood ratio (3.91) and negative likelihood ratio (0.68). Based on this analysis of the score, in positive cases (score > 6) the infants had nearly a fourfold greater chance (3.91) of presenting lung disease.

Based on this analysis, the scores were divided into two categories (≤ 6 and > 6). CT scans with scores greater than six contained numerous abnormalities, while those with scores less than or equal to six either contained few abnormalities or were normal. Subsequent analysis on the scores in two categories showed that the majority of the patients (64 newborns) scored from zero to six; of these, 36 patients (NPV of 56.3%) did not present respiratory morbidity during the first year. Scores higher than six were found in 22 patients and, of these patients, 40.9% (PPV) presented wheezing, 45.5% (PPV) hospitalizations and 63.6% (PPV) pneumonia. Table 3 shows the validity measurements of the scores in relation to respiratory morbidity.

**Table 3.** Validity measurements for scores greater than six in relation to wheezing, pneumonia, hospitalization and respiratory morbidity during the first year of life

Respiratory morbidity	Sensitivity % (95% CI)	Specificity % (95% CI)	PPV % (95% CI)	NPV % (95% CI)
Wheezing	40.9	79.7	40.9	79.7
	(20.4-61.5)	(69.8-89.5)	(20.4-61.5)	(69.8-89.5)
Pneumonia	43.8	85.2	63.6	71.9
	(26.6-60.9)	(75.7-94.7)	(43.5-83.7)	(60.9-82.9)
Hospitalization	45.5	81.3	45.5	81.3
	(24.6-66.3)	(71.7-90.8)	(24.6-66.3)	(71.7-90.8)
Respiratory morbidity	39.1	90.0	81.8	56.3
	(25.0-53.2)	(80.7-99.3)	(65.7-97.9)	(44.1-68.4)

PPV = positive predictive value; NPV = negative predictive value; CI = confidence interval.

This cutoff point (> 6) was also evaluated in relation to diagnoses of BPD that were made during the neonatal period. Among the children with scores greater than six, 59.1% had BPD. Among the patients with scores less than six, 81.3% did not have BPD.

As in other hospital-based studies, a convenience sample was used. The estimators of predictive values were accompanied by their respective confidence intervals, which made it possible to achieve statistical inferences and evaluate the precision. However, another way to statistically evaluate the predictive ability of tomographic scores higher than six (HRCT with greater numbers of abnormalities) would be to retrospectively investigate the power of the study. This analysis on the study power was performed by setting the chance of committing a type I error (alpha) as 5%. The complement of type II (beta) error, i.e. the power (1-beta) varied according to the magnitude of the proportions found and their differences. The power of this study was found to range between 86 and 96% for respiratory morbidity and for the separate variables of each respiratory complication, except for hospitalization, which showed a test power of 51%. Thus, only the estimators relating to hospitalization would need a larger sample (n = 106 patients).

## DISCUSSION

Among the few studies in the literature on HRCT scans among premature infants,^2,5-11,17^ only three^2,3,11^ analyzed CT scans performed exclusively in the neonatal period. Moreover, as far as we can ascertain, only Ochiai et al.^11^ presented a scoring system to evaluate pulmonary involvement based on CT abnormalities in tests performed so early in life.

Studies have shown that children who developed BPD frequently presented signs of chronic lung disease. The CT findings were hyperaeration, linear opacities, triangular subpleural opacities and bronchovascular distortion or thickening.^10,11,17^ The presence of these abnormalities and absence of bronchiectasis suggested that these were sequelae of BPD.^10^ Pulmonary hyperaeration was the most frequent abnormality and correlated significantly with clinical scores, with a high interobserver agreement.^17^ HRCT scans on patients ranging in age from five to 18 years who were born prematurely and had a history of BPD, showed a significant correlation with abnormal lung function.^6^

In 2007, Mahut et al.^8^ described the CT abnormalities found on the scans of 41 very low birth weight premature patients, all with BPD. The scans were performed between 10 and 20 months after birth. These authors concluded that despite advances in perinatal care, the CT abnormalities currently found were still similar to those that used to be described in the pre-surfactant era and were associated with duration of oxygen therapy and mechanical ventilation, while emphasizing that they did not detect the bronchial involvement that had been described in previous studies.

Ochiai et al.^11^ analyzed HRCT scans on 42 premature infants, all with a diagnosis of BPD, whose scans were performed when they were clinically stable and with an age range similar to that of our study. They developed a scoring system based on a radiographic score for BPD with three groups of abnormalities (hyperaeration, emphysema and fibrous or interstitial abnormalities) and correlated this system with clinical scores that were assigned at the corrected ages of 28 days and 36 weeks.

The principal difference between the scoring system of Ochiai et al.^11^ and the system that we are proposing is that we evaluated patients both with and without BPD, which thus allowed us to conclude that CT abnormalities like air trapping, atelectasis, consolidation, ground-glass opacity and air bubbles are not characteristic of BPD. These CT findings are present in premature infants who are exposed to mechanical ventilation and oxygen therapy, with or without clinical criteria for a diagnosis of BPD. According to our findings, these abnormalities are more prominent in patients that developed BPD and those with respiratory morbidity during the first year of life.

The most frequent single pulmonary lesions demonstrated by HRCT scans in our study were atelectasis and ground-glass opacity. Other authors^6-8,17^ observed high frequencies of these same lesions, but since they mostly examined older patients with images obtained under conditions of apnea and with series during inspiration and expiration, they showed a higher frequency of air trapping than seen in our study. The immaturity of the lungs of premature infants, associated with the complications caused by the oxygen therapy and mechanical ventilation needed to keep them alive, can lead to decreased pulmonary compliance and modifications to the pulmonary architecture, which are expressed on HRCT scans by these frequently identified lesions.

One of the limitations of our study was the fact that the CT scans were performed on young, very small patients with elevated respiratory rates. Because of the young age and thus the lack of collaboration by the patients, it was not possible to perform the test under conditions of apnea. Thus, artifacts are known to occur that are secondary to the newborns’ typical movements and their respiratory pattern. Such artifacts might be resolved if the child received general anesthesia and mechanical ventilation, which would make it possible to obtain CT images under conditions of apnea. Respiratory movements hinder the detailed evaluation of abnormalities like ground-glass opacity and air trapping. These factors contributed towards lower interobserver agreement for these two lesions. Nonetheless, we believe that since CT is an invasive procedure, and given the labile clinical conditions in the majority of these newborns, performing the test under general anesthesia should be avoided.

Another possible limitation to our study was the acquisition time required for the CT images, which contributed with higher doses of radiation and increased secondary artifacts in the premature infants’ respiratory patterns. At the time when the scans were performed, the CT equipment that was used did not allow the technical settings to be changed to make the time taken and thus the radiation dose used as low as possible under the circumstances. Therefore, the settings used were higher than those proposed by Lucaya et al.^33^ in 2000 (35 to 60 mAs), but similar to those used recently by Mahut et al.^8^ in equipment equivalent to ours. The latter authors performed HRCT scans on premature infants born between January 1999 and March 2001, with settings of 100 mA and 1.0 s.

Although the age of these newborns tends to decrease the quality of some CT images, we believe that this should not be viewed as an impediment to performing this test, which proved important for evaluating the extent of pulmonary involvement. Ochiai et al.^11^ concluded that their scoring system was capable of assessing the patient's clinical status before hospital discharge and predicting the prognosis of patients with BPD, but they did not evaluate the predictive validity of the score as was done in our study.

Another important factor in performing HRCT scans on such young patients was demonstrated by the score, which showed low sensitivity but high specificity, thereby indicating that a large portion of the patients without respiratory morbidity during the first year of life presented tests that were normal or only presented limited abnormalities. Meanwhile, the PPV was high, thus indicating a chance of more than 80% that the infants with abnormal scans would present respiratory morbidity during the first year of life. Importantly, in tertiary and reference centers, HRCT is a valid method for use in the late neonatal period because of the very high prevalence of respiratory morbidity (56.2%) seen in the follow-ups on this high-risk population.^2^ There needs to be a mechanism for indicating which patients are most likely to become ill, and therefore, investments in prevention with vaccines and other expensive therapies are necessary. However, radiologists need to be trained to become familiar with the types of pulmonary abnormalities identified using HRCT on this group of very small patients that present impairment according to the scoring system proposed here.

The purpose of this new scoring system was to estimate the likelihood of respiratory morbidity during the first year of life among children with abnormal HRCT scans, as was demonstrated by the predictive values for the CT score, i.e. good positive predictive value and high specificity. The assessment on the HRCT scoring system further demonstrated that it should not be used for screening purpose because of the high percentage of false negatives (44%). The CT score sensitivity and specificity showed that HRCT scans should not be indicated in hospitals and services where the prevalence of prematurity and respiratory morbidity could be low, because the PPV would be very low.

## CONCLUSIONS

The scoring system that we propose proved to be reproducible and easy to apply. We thus conclude that this CT scoring system is important in clinical practice, since it allows standardization of the evaluation of CT abnormalities in premature infants, through identifying patients with increased likelihood of respiratory morbidity during the first year of life. Furthermore, by expanding the use of this scoring system, CT scans on premature infants with different risk factors for respiratory morbidity can be compared. Scores greater than six showed a capacity to predict respiratory morbidity in 81.8% of patients, but the sensitivity of the score was 39.1%, which means that out of 100 patients with respiratory morbidity during the first year of life, only 39 patients presented scores greater than six.
